# Quantifying the ‘distance to LDC-goal’ in patients at very high cardiovascular risk with hyperlipidaemia in Germany: a retrospective claims database analysis

**DOI:** 10.1177/17539447241277402

**Published:** 2024-09-28

**Authors:** Ksenija Stach, Hartmut Richter, Uwe Fraass, Alexandra Stein

**Affiliations:** 5th Medical Department, University Hospital Mannheim, Heidelberg University, Theodor-Kutzer-Ufer 1-3, Mannheim 68167, Germany; IQVIA Epidemiology, IQVIA, Frankfurt/Main, Germany; Amgen GmbH, Munich, Germany; Amgen GmbH, Munich, Germany

**Keywords:** cardiovascular risk, LDL-C goals, LDL-C reduction, LDL-C target, lipid-lowering therapy, low-density lipoprotein cholesterol

## Abstract

**Background and objectives:**

This study quantified the ‘distance to LDL-C goal’ in patients at very high cardiovascular risk with uncontrolled hyperlipidaemia. ‘Distance to LDL-C goal’ was defined as the percentage by which low-density lipoprotein cholesterol (LDL-C) levels needed to be reduced to achieve the LDL-C goals specified in the 2016 or 2019 European Society of Cardiology/European Atherosclerosis Society guidelines.

**Design and methods:**

This retrospective analysis using data from the IQVIA Disease Analyzer database included patients who were predominantly treated by a primary care physician, diabetologist or cardiologist between 2014 and 2018, with a diagnosis of hyperlipidaemia and an initial LDL-C measurement (index event) and one or more cardiovascular risk factors. The primary outcome was to assess the proportion of patients with uncontrolled hyperlipidaemia and to classify the ‘distance to LDL-C goal’ in these patients.

**Results:**

Data from 32,963 patients were analysed (*n* = 27,159, *n* = 3873 and *n* = 1931 patients in the primary care physician, diabetology and cardiology cohorts, respectively). Most patients had uncontrolled LDL-C levels (⩾70 mg/dL; ⩾1.8 mmol/L) at index (91.0%, 86.4% and 94.0% of patients in the primary care physician, diabetology and cardiology cohorts, respectively). Analysis of the ‘distance to LDL-C goal’ indicated that approximately one-third of patients in each cohort required an LDL-C level reduction of up to 50% relative to index to achieve their LDL-C goal (35.8%, 43.7% and 28.4% of patients in the primary care physician, diabetology and cardiology cohorts, respectively). LDL-C control was not achieved at 36 months post-index in most patients with uncontrolled LDL-C levels (86.8%, 81.7% and 90.2% of patients in the primary care physician, diabetology and cardiology cohorts, respectively).

**Conclusion:**

LDL-C levels were uncontrolled in most patients with hyperlipidaemia. Analysis of the ‘distance to LDL-C goal’ showed that most patients required a substantial LDL-C level reduction to achieve their LDL-C goal.

## Introduction

Cardiovascular disease (CVD), including atherosclerotic CVD (ASCVD), is the leading cause of death worldwide.^1^ In the European Union, CVD accounts for 37% of all deaths.^2^ The burden of CVD is growing rapidly owing to the prevalence of major risk factors, such as smoking, obesity, hypertension and hyperlipidaemia.^3,4^ Notably, hyperlipidaemia is a risk factor for both fatal and non-fatal cardiovascular (CV) events, owing to the high levels of low-density lipoprotein cholesterol (LDL-C) and low levels of high-density lipoprotein cholesterol in the blood.^5^ LDL-C levels are causally related to ASCVD and its progression; thus, the lowering of LDL-C levels is required to reduce the risk of CV events.^6,7^ Previous studies have demonstrated that the effect of LDL-C levels on the risk of ASCVD is determined by the absolute magnitude and length of exposure to high LDL-C levels.^7,8^ Accordingly, early and long-term reductions in LDL-C levels significantly decrease the risk of CV events.^9^

Strategies for the prevention and management of CV events focus on improving lifestyle factors^10^ and the use of lipid-lowering therapies (LLTs) to reduce LDL-C levels.^8,11^ The 2019 European Society of Cardiology/European Atherosclerosis Society (ESC/EAS) guidelines recommend treatment with high-intensity statins for patients at very high CV risk to achieve an LDL-C goal of less than 55 mg/dL (<1.4 mmol/L) and an LDL-C level reduction of at least 50% from baseline.^7^ Add-on treatment with a non-statin LLT (such as ezetimibe) and/or a proprotein convertase subtilisin/kexin type 9 (PCSK9) inhibitor (such as evolocumab or alirocumab) is also recommended to supplement a maximally tolerated statin dose.^7^ For individuals with statin intolerance, non-statin LLTs are also indicated as a monotherapy.^7^ In the previous 2016 ESC/EAS guidelines, an LDL-C goal of less than 70 mg/dL (<1.8 mmol/L) was recommended.^12^ Studies have shown that the intensification of LLTs (such as the addition of ezetimibe or PCSK9 inhibitors to statins) can reduce LDL-C levels by approximately 25%–60% and also lower the risk of CV events.^13–19^ Thus, for patients at very high CV risk, treatment intensification may be required to achieve their LDL-C goals.^7,13^

Real-world evidence from claims data examining target attainment for LLT in different patient populations with hyperlipidaemia is limited.^20,21^ The existing real-world evidence data are also predominantly dichotomous, in that the data only describe whether patients achieved or did not achieve their LDL-C goal. This real-world study used electronic medical records to assess the proportion and distribution of patients in Germany with hyperlipidaemia and a very high CV risk who did not achieve their LDL-C goals. The focus of this study was to quantify the ‘distance to LDL-C goal’ in these patients (i.e. the degree to which LDL-C levels needed to be reduced to achieve LDL-C goals, according to the 2016 or 2019 ESC/EAS guidelines).

## Patients and methods

### Study design

This study was a retrospective analysis of the IQVIA Disease Analyzer database of anonymized patient data and used the electronic medical records of patients treated outside of the hospital setting. Data were collected from the routine medical documentation of more than 2500 practices, with more than 3100 physicians. The overall study period was from January 2013 to September 2021. An index date (i.e. the date of the initial LDL-C measurement) was defined per patient in the period from January 2014 to December 2018, and each patient was observed for a period ranging from 24 months (12 months both pre-index and post-index) to 48 months (12 months pre-index and 36 months post-index) surrounding this date. The first LDL-C test result and its recorded date were the index event and the index date, respectively.

### Patient eligibility

Patients aged 18 years or older were included in the study if they satisfied specific criteria. Patients must have had an initial LDL-C laboratory test result between January 2014 and December 2018. A diagnosis of hyperlipidaemia (third level ICD-10: E78 including all fourth level subcodes except E78.1 and E78.3) on the index date or in the 12 months before the index date and at least one additional ASCVD risk factor or established CV condition (defined as myocardial infarction, ischaemic stroke, transient ischaemic attack, coronary ischaemic heart disease, peripheral arterial disease, coronary bypass, percutaneous coronary intervention (angioplasty)) on the index date or in the 12 months before the index date was required for inclusion, meaning that all eligible patients were considered to have a very high CV risk. At least one additional LDL-C test result in the first 12 months (32–365 days) after the index date and patient observability in the database available for at least 12 months before the index date and at least 12 months after the index date were also necessary for inclusion. Patients were excluded from the study if the practice visited by the patient did not supply data continuously in the 24 months surrounding the index date (12 months both pre-index and post-index).

### Outcomes

The first primary objective was the proportion of patients with hyperlipidaemia and a very high CV risk who achieved their LDL-C goal of less than 70 mg/dL (<1.8 mmol/L) as per the 2016 ESC/EAS guidelines. The 2016 ECS/EAS guideline treatment target was used in preference to the more recent 2019 ESC/EAS guideline target because the index dates of all patients were recorded prior to 2019. The treatment goal of LDL-C levels of less than 70 mg/dL (<1.8 mmol/L) was consistent with the older 2012 ESC/EAS guidelines, which may have been in effect at index date for some patients. The relative goal of at least a 50% reduction from baseline LDL-C was not assessed in the current study. The second primary objective was quantification of the ‘distance to LDL-C goal’ in patients with uncontrolled LDL-C status at index. ‘Distance to LDL-C goal’ was defined as the LDL-C reduction required to meet a guideline threshold. The reduction from index was based on the ‘distance to LDL-C goal’ rather than an absolute LDL-C level. Patients with controlled status were defined as those who had achieved their LDL-C goals.

An analysis of sociodemographic and comorbidity status, including baseline characteristics, LLT use, CV risk factors and established CVDs, in patients with controlled or uncontrolled hyperlipidaemia was a secondary objective. Additionally, the factors associated with the probability of being controlled or uncontrolled at index, quantification of the time taken to achieve a controlled status and factors associated with becoming controlled post-index were also analysed as secondary objectives.

Sensitivity analyses were conducted on data from patients with uncontrolled LDL-C levels at index who had an LDL-C measurement that was at least 25% above their goal. This threshold was assigned as a measure of clinical relevance.

All results were classified by cohort of the speciality of the treating physician: (1) primary care physician cohort; (2) diabetology cohort (primary care physicians with additional diabetology or German Diabetes Society (Deutsche Diabetes Gesellschaft) training); or (3) cardiology cohort (cardiologists). The results were also classified according to the patients’ lipid levels (i.e. controlled or uncontrolled LDL-C status).

### Statistical analysis

The endpoints were analysed descriptively: distributions for categorical variables, statistics for continuous variables (mean and standard deviation) and Kaplan–Meier curves for time-to-event outcomes. Hazard ratios (HRs) for the time-stratified probability of reaching control were estimated using a Cox regression analysis. Odds ratios for the probability of reaching control were calculated using logistic regression.

### Ethical conduct

Given that the study was based on retrospective de-identified data, ethical approval was not required.

## Results

### Patient characteristics

In total, data for 315,271 patients with known index dates (*n* = 273,086 in the primary care physician cohort, *n* = 30,608 in the diabetology cohort and *n* = 11,577 in the cardiology cohort) were stored within the database. After the implementation of the inclusion and exclusion criteria, data were obtained from 27,159, 3873 and 1931 patients in the primary care physician, diabetology and cardiology cohorts, respectively. Overall, women accounted for 40.4% (*n* = 10,964) of the primary care physician cohort, 39.1% (*n* = 1515) of the diabetology cohort and 37.1% (*n* = 717) of the cardiology cohort. Controlled status at index was more common in men than in women. In each cohort, the mean patient age was similar between patients with controlled and uncontrolled status at index. Patients aged 80 years and older represented 20.6% (*n* = 5605) of the primary care physician cohort, 20.0% (*n* = 776) of the diabetology cohort and 10.4% (*n* = 200) of the cardiology cohort. Among the included CV risk factors or established CVDs, chronic ischaemic heart disease was most prevalent in all three cohorts: 74.2% (*n* = 20,144) in the primary care physician cohort, 68.6% (*n* = 2658) in the diabetology cohort and 90.4% (*n* = 1745) in the cardiology cohort (Table 1 and Supplemental Table 1).

**Table 1.** Baseline patient characteristics by speciality cohort.

Parameter	Primary care physician cohort			Diabetology cohort			Cardiology cohort		
	Overall	Controlled	Uncontrolled	Overall	Controlled	Uncontrolled	Overall	Controlled	Uncontrolled
Total patients	27,159 (100.0)	2449 (9.0)	24,710 (91.0)	3873 (100.0)	525 (13.6)	3348 (86.4)	1931 (100.0)	116 (6.0)	1815 (94.0)
Sex									
Female	10,964 (40.4)	668 (27.3)	10,296 (41.7)	1515 (39.1)	138 (26.3)	1377 (41.1)	717 (37.1)	23 (19.8)	694 (38.2)
Age									
Mean (SD)	71.4 (10.8)	71.7 (11.0)	71.4 (10.8)	71.6 (10.5)	71.5 (10.7)	71.6 (10.5)	68.9 (10.0)	70.1 (10.5)	68.8 (10.0)
18–50 years	1019 (3.7)	93 (3.8)	926 (3.7)	125 (3.2)	20 (3.8)	105 (3.1)	88 (4.5)	7 (6.0)	81 (4.5)
> 50–60 years	3658 (13.5)	330 (13.5)	3328 (13.5)	500 (12.9)	75 (14.3)	425 (12.7)	312 (16.2)	14 (12.1)	298 (16.4)
> 60–70 years	6644 (24.5)	571 (23.3)	6073 (24.6)	962 (24.8)	114 (21.7)	848 (25.3)	579 (30.0)	32 (27.6)	547 (30.1)
> 70–80 years	10,233 (37.7)	909 (37.1)	9324 (37.7)	1510 (39.0)	212 (40.4)	1298 (38.8)	752 (38.9)	43 (37.1)	709 (39.1)
> 80 years	5605 (20.6)	546 (22.3)	5059 (20.5)	776 (20.0)	104 (19.8)	672 (20.1)	200 (10.4)	20 (17.2)	180 (9.9)
CVDs									
Myocardial infarction	3924 (14.4)	518 (21.2)	3406 (13.8)	352 (9.1)	69 (13.1)	283 (8.5)	235 (12.2)	23 (19.8)	212 (11.7)
Ischaemic stroke	6736 (24.8)	640 (26.1)	6096 (24.7)	969 (25.0)	109 (20.8)	860 (25.7)	430 (22.3)	35 (30.2)	395 (21.8)
Transient ischaemic attack	2352 (8.7)	186 (7.6)	2166 (8.8)	278 (7.2)	27 (5.1)	251 (7.5)	33 (1.7)	1 (0.9)	32 (1.8)
Coronary bypass	1565 (5.8)	182 (7.4)	1383 (5.6)	281 (7.3)	49 (9.3)	232 (6.9)	272 (14.1)	20 (17.2)	252 (13.9)
PCI (angioplasty) with stenting	1819 (6.7)	232 (9.5)	1587 (6.4)	404 (10.4)	80 (15.2)	324 (9.7)	485 (25.1)	48 (41.4)	437 (24.1)
Carotid artery stenosis	2007 (7.4)	204 (8.3)	1803 (7.3)	277 (7.2)	42 (8.0)	235 (7.0)	182 (9.4)	15 (12.9)	167 (9.2)
Angina pectoris	3684 (13.6)	341 (13.9)	3343 (13.5)	291 (7.5)	53 (10.1)	238 (7.1)	955 (49.5)	18 (15.5)	937 (51.6)
PAD	5271 (19.4)	458 (18.7)	4813 (19.5)	1104 (28.5)	177 (33.7)	927 (27.7)	172 (8.9)	21 (18.1)	151 (8.3)
CAD^a^	20,708 (76.2)	1974 (80.6)	18,734 (75.8)	2719 (70.2)	397 (75.6)	2322 (69.4)	1756 (90.9)	108 (93.1)	1648 (90.8)
CVD^b^	7894 (29.1)	753 (30.7)	7141 (28.9)	1134 (29.3)	134 (25.5)	1000 (29.9)	568 (29.4)	44 (37.9)	524 (28.9)
CAD and CVD	3903 (14.4)	410 (16.7)	3493 (14.1)	515 (13.3)	75 (14.3)	440 (13.1)	410 (21.2)	38 (32.8)	372 (20.5)
CAD and PAD	2987 (11.0)	302 (12.3)	2685 (10.9)	559 (14.4)	101 (19.2)	458 (13.7)	140 (7.3)	16 (13.8)	124 (6.8)
CAD or CVD or PAD	26,372 (97.1)	2416 (98.7)	23,856 (96.9)	3790 (97.9)	521 (99.2)	3269 (97.6)	1927 (99.8)	116 (100.0)	1811 (99.8)
CAD and CVD and PAD	972 (3.6)	96 (3.9)	876 (3.5)	158 (4.1)	23 (4.4)	135 (4.0)	59 (3.1)	10 (8.6)	49 (2.7)
Cardiovascular risk factors									
Metabolic syndrome	1380 (5.1)	110 (4.5)	1270 (5.1)	167 (4.3)	23 (4.4)	144 (4.3)	16 (0.8)	1 (0.9)	15 (0.8)
CKD and diabetes mellitus	2506 (9.2)	269 (11.0)	2237 (9.1)	848 (21.9)	148 (28.2)	700 (20.9)	57 (3.0)	3 (2.6)	54 (3.0)

Data presented as *n* (%) unless stated otherwise. Controlled status was defined as those who had achieved their LDL-C goals; uncontrolled status was defined as those who had not achieved their LDL-C goals. ^a^CAD was defined as myocardial infarction and/or heart failure (acute or chronic). ^b^CVD was defined as stroke and/or carotid artery stenosis.

CAD, coronary artery disease; CKD, chronic kidney disease; CVD, cardiovascular disease; LDL-C, low-density lipoprotein cholesterol; PAD, peripheral arterial disease; PCI, percutaneous coronary intervention; SD, standard deviation.

### LLT use at index

The proportions of patients not receiving any LLT at index were 39.9% (*n* = 10,843), 56.6% (*n* = 2194) and 75.5% (*n* = 1457) for patients in the primary care physician, diabetology and cardiology cohorts, respectively. Of the patients who had controlled LDL-C levels at index, 16.3% (*n* = 400), 48.8% (*n* = 256) and 77.6% (*n* = 90) of patients in the primary care physician, diabetology and cardiology cohorts, respectively, reported not receiving any LLT. Of those with uncontrolled LDL-C levels at index, 42.3% (*n* = 10,443), 57.9% (*n* = 1938) and 75.3% (*n* = 1367) of patients in the primary care physician, diabetology and cardiology cohorts, respectively, reported not receiving any LLT (Supplemental Table 2).

Across all cohorts, statins were the most frequently administered LLT at index, followed by statins with ezetimibe. In the primary care cohort, the proportions of patients who received moderate-intensity and high-intensity statin monotherapy were lower in the uncontrolled group than in those who had controlled LDL-C levels at index (moderate-intensity: 42.6% (*n* = 10,538) vs 58.9%, (*n* = 1443) respectively; high-intensity: 6.8% (*n* = 1678) vs 14.0% (*n* = 342), respectively; Supplemental Table 2).

The assessment of LLT post-index in patients who had uncontrolled LDL-C levels at index and reached control is described in Supplemental Table 3. The proportions of patients who did not receive any LLT after the index date despite not having met the target levels of LDL-C per the 2016 ESC/EAS guidelines were 19.7% (*n* = 4785), 26.6% (*n* = 878) and 41.8% (*n* = 708) in the primary care physician, diabetology and cardiology cohorts, respectively.

### Distribution of LDL-C levels for patients uncontrolled at index

**Figure 1.**
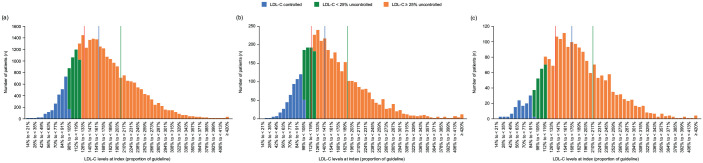
Distributions of LDL-C levels at index. (a) Primary care physician cohort (*N* = 27,159), (b) diabetology cohort (*N* = 3873), (c) cardiology cohort (*N* = 1931) – primary analysis. Lines denote the quartiles to the overall distribution (red: lower quartile, blue: median, green: upper quartile). Controlled status was defined as those who had achieved their LDL-C goals, uncontrolled status was defined as those who had not achieved their LDL-C goals. The green bars represent patients who were uncontrolled but were not included in the sensitivity analysis because the sensitivity analysis required patients to be at least 25% above the goal. LDL-C, low-density lipoprotein cholesterol.

Most patients had uncontrolled LDL-C levels (⩾70 mg/dL; ⩾1.8 mmol/L) at index: 91.0% (*n* = 24,710), 86.4% (*n* = 3348) and 94.0% (*n* = 1815) of patients in the primary care physician, diabetology and cardiology cohorts, respectively (Table 1). The distributions of LDL-C levels at index in each cohort are reported in Figure 1. The three cohorts exhibited a similar distribution of LDL-C levels at index.

### Quantification of the ‘distance to LDL-C goal’ in patients uncontrolled at index

**Figure 2.**
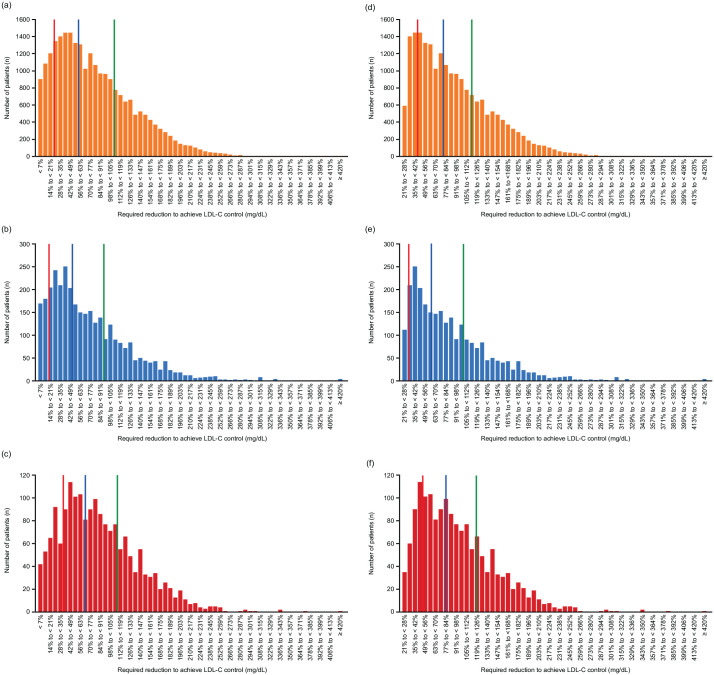
Distribution of LDL-C levels of the ‘distance to goal’: the minimum LDL-C reduction required for patients uncontrolled at index to achieve control. (a) Primary care physician cohort (*N* = 24,710), (b) diabetology cohort (*N* = 3348), (c) cardiology cohort (*N* = 1815) – primary analysis. (d) Primary care physician cohort (*N* = 20,755), (e) diabetology cohort (*N* = 2662), (f) cardiology cohort (*N* = 1598) – sensitivity analysis. Lines denote the quartiles (red: lower quartile, blue: median, green: upper quartile). LDL-C, low-density lipoprotein cholesterol.

The overall distributions of the minimum LDL-C reductions required for patients who had uncontrolled LDL-C levels at index to achieve control were similar among the cohorts (Figure 2(a)–(c)). Approximately one-third of patients in each cohort required an LDL-C level reduction of up to 50% relative to index to achieve their LDL-C goal (35.8% (*n* = 8841), 43.7% (*n* = 1462) and 28.4% (*n* = 516) of patients in the primary care physician, diabetology and cardiology cohorts, respectively). Approximately one-third of patients in each cohort required an LDL-C level reduction of between 50% and 100% relative to index to achieve control (31.8% (*n* = 7866), 29.2% (*n* = 977) and 35.1% (*n* = 637) of patients in the primary care physician, diabetology and cardiology cohorts, respectively). Approximately one-fifth of patients required an LDL-C level reduction of between ~100% and 150% relative to index to reach their LDL-C goals (19.1% (*n* = 4727), 16.4% (*n* = 548) and 22.5% (*n* = 408) of patients in the primary care physician, diabetology and cardiology cohorts, respectively). Approximately one-tenth of patients required an LDL-C level reduction of between ~150% and 200% relative to index to reach their LDL-C goals. A small proportion of patients required a minimum reduction of 200% relative to index to reach their LDL-C goals (3.8% (*n* = 942), 3.7% (*n* = 123) and 4.2% (*n* = 76) of patients in the primary care physician, diabetology and cardiology cohorts, respectively). In the sensitivity analysis, similar results were observed in patients who were uncontrolled and who had an LDL-C measurement at index that was at least 25% above their LDL-C goals, as in the primary analysis (Figure 2(d)–(f)).

### Factors associated with LDL-C control at index

The results of the logistic regression analyses for factors associated with control status at index are presented in Table 2 and Supplemental Table 4. LLT status at index was associated with LDL-C control, particularly in the primary care physician and diabetology cohorts, in which statin therapy was significantly associated with LDL-C control in a dose-dependent manner (Table 2). There were no significant associations between treatment with statins and LDL-C control at index in patients in the cardiology cohort. In all cohorts, female sex was associated with a significantly lower likelihood of LDL-C control at index (*p* value: <0.0001, <0.0001 and 0.0151 for the primary care physician, diabetology and cardiology cohorts, respectively). Across all cohorts, diabetes mellitus was significantly associated with an increased likelihood of LDL-C control at index (Table 2). Several CV risk factors were associated with an increased likelihood of LDL-C control at index. In the primary care physician cohort, myocardial infarction, diabetes mellitus, ischaemic stroke and heart failure were significantly associated with an increased likelihood of LDL-C control at index (Table 2 and Supplemental Table 4). In the diabetology cohort, myocardial infarction, diabetes mellitus and percutaneous coronary intervention (angioplasty) with stenting were significantly associated with an increased likelihood of LDL-C control at index (Table 2). In the cardiology cohort, percutaneous coronary intervention (angioplasty) with stenting, angina pectoris and diabetes mellitus were significantly associated with an increased likelihood of LDL-C control at index (Table 2). Similar results for each cohort were observed in the sensitivity analysis (Supplemental Table 5).

**Table 2.** Results of the logistic regression analyses for the odds of being controlled (yes/no) for the various covariate parameters.

Parameter	Primary care physician cohort		Diabetology cohort		Cardiology cohort	
	Odds ratio (95% CI)	*p* value	Odds ratio (95% CI)	*p* value	Odds ratio (95% CI)	*p* value
Sex (ref: male)						
Female	0.578 (0.525–0.638)	<0.0001	0.550 (0.442–0.683)	<0.0001	0.534 (0.321–0.886)	0.0151
Age class (ref: >60–70 years)						
18–30 years	2.851 (0.58–14.013)	0.1972	NA	NA	NA	NA
>30–40 years	0.834 (0.396–1.755)	0.6327	0.541 (0.071–4.133)	0.5539	3.092 (0.546–17.495)	0.2018
>40–50 years	0.987 (0.769–1.266)	0.918	1.725 (0.991–3.000)	0.0538	1.560 (0.565–4.31)	0.3912
>50–60 years	0.979 (0.845–1.133)	0.7735	1.317 (0.953–1.819)	0.0953	0.833 (0.42–1.654)	0.6022
>70–80 years	1.097 (0.98–1.229)	0.1087	1.264 (0.983–1.626)	0.0677	0.925 (0.56–1.53)	0.7628
>80 years	1.357 (1.19–1.547)	<0.0001	1.310 (0.971–1.768)	0.0774	2.237 (1.173–4.268)	0.0145
Cardiovascular risk factors						
Myocardial infarction	1.303 (1.166–1.455)	<0.0001	1.430 (1.062–1.924)	0.0184	1.472 (0.862–2.513)	0.1566
Ischaemic stroke	1.133 (1.016–1.264)	0.0249	0.917 (0.712–1.182)	0.5047	1.139 (0.695–1.865)	0.6066
Transient ischaemic attack	0.934 (0.793–1.1)	0.4153	0.809 (0.53–1.233)	0.3239	0.435 (0.056–3.386)	0.4268
Peripheral arterial disease	0.910 (0.811–1.021)	0.109	1.205 (0.964–1.507)	0.1018	1.524 (0.87–2.673)	0.141
Cardiac bypass	1.000 (0.847–1.182)	0.9963	1.061 (0.755–1.492)	0.7328	1.055 (0.609–1.828)	0.8485
PCI (angioplasty) with stenting	1.141 (0.981–1.328)	0.0872	1.427 (1.078–1.888)	0.0129	1.705 (1.108–2.624)	0.0154
Carotid artery stenosis	1.030 (0.879–1.208)	0.7131	1.138 (0.795–1.627)	0.4807	1.441 (0.763–2.72)	0.2604
Angina pectoris	0.907 (0.8–1.028)	0.1249	1.295 (0.929–1.804)	0.1269	0.214 (0.124–0.369)	<0.0001
Diabetes mellitus	1.474 (1.35–1.61)	< 0.001	1.996 (1.572–2.536)	<0.0001	2.743 (1.793–4.194)	<0.0001
Chronic kidney disease	1.049 (0.93–1.183)	0.4364	1.200 (0.966–1.492)	0.100	0.778 (0.371–1.63)	0.506
Metabolic syndrome	0.810 (0.658–0.998)	0.0482	0.858 (0.539–1.365)	0.5174	0.359 (0.04–3.207)	0.3592
Statin dose at index (ref: no statin therapy)						
Low-intensity statins	1.680 (1.296–2.178)	<0.0001	0.641 (0.306–1.344)	0.2391	<0.001 (<0.001–>999.999)	0.9783
Moderate-intensity statins	3.240 (2.891–3.631)	<0.0001	1.543 (1.253–1.901)	<0.0001	0.916 (0.516–1.628)	0.7658
High-intensity statins	4.803 (4.126–5.591)	<0.0001	1.975 (1.407–2.771)	<0.0001	0.661 (0.222–1.966)	0.4565
Ezetimibe at index (ref: no ezetimibe)						
Statins with ezetimibe	1.642 (1.385–1.947)	<0.0001	1.266 (0.796–2.012)	0.3189	1.766 (0.673–4.629)	0.2478
PCSK9 inhibitor at index (ref: no PCSK9 inhibitor)						
Statins with PCSK9 inhibitor	< 0.001 (<0.001–>999.999)	0.9413	NA	NA	4.831 (0.779–29.943)	0.0906
Other LLT^a^ at index (ref: no other LLT)						
Statins with other LLT^a^	1.109 (0.647–1.901)	0.7061	0.387 (0.089–1.689)	0.2069	<0.001 (<0.001–>999.999)	0.9916
Statin tolerance (ref: statin tolerant)						
Statin intolerant	0.424 (0.2–0.902)	0.026	1.206 (0.5–2.909)	0.6761	2.245 (1.183–4.259)	0.0133

A multivariable model was used for these analyses.

a Other LLT = fibrates or bile acid sequestrants.

CI, confidence interval; LLT, lipid-lowering therapy; NA, not available; PCI, percutaneous coronary intervention; PCSK9, proprotein convertase subtilisin/kexin type 9; ref, reference.

### Time-to-LLT intensification and time-to-LDL-C control

**Figure 3.**
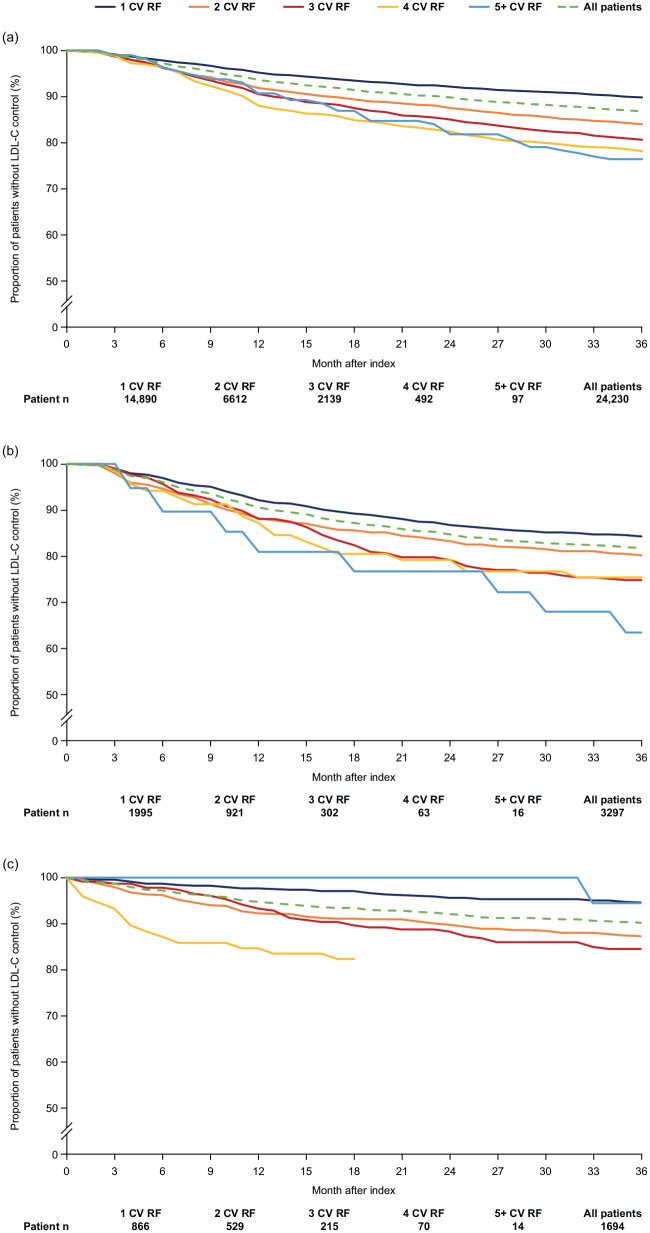
Kaplan–Meier curves for time-to-LDL-C control among patients with uncontrolled LDL-C at index. (a) Primary care physician cohort, (b) diabetology cohort, (c) cardiology cohort. Data are presented overall and by number of CV risk factors or CV diseases. Y-axis is offset at 50% to show the differences between the curves more clearly. Curves do not decline sufficiently to be able to determine the quartiles. Data for the 4 CV RF group comprising censored patients after 18 months. CV, cardiovascular; LDL-C, low-density lipoprotein cholesterol; RF, risk factor.

The proportions of patients who were uncontrolled at index without LLT escalation at 36 months post-index were 66.6% in the primary care physician cohort, 59.8% in the diabetology cohort and 59.0% in the cardiology cohort (Supplemental Figure 1). Kaplan–Meier analyses revealed that LDL-C control was not achieved in most patients at 36 months post-index (86.8%, 81.7% and 90.2% of patients in the primary care physician, diabetology and cardiology cohorts, respectively; Figure 3). Stratification of patients by the number of CV risk factors or established CV conditions revealed an association between the number of CV risk factors or established CV conditions and the time-to-LDL-C control. A lower probability of LDL-C control at 36 months post-index was seen among patients with one CV risk factor or CVD than among those with five or more CV risk factors or CVDs (except in the cardiology cohort owing to low patient counts; Figure 3).

A Cox regression analysis indicated the superiority of high-intensity statin treatments at index in increasing the probability of achieving LDL-C control after the index date (Supplemental Table 6). Moderate-intensity statins were significantly associated with LDL-C control post-index (HR vs no statins: 2.63 in the primary care physician cohort (*p* < 0.0001), 2.02 in the diabetology cohort (*p* < 0.0001) and 1.98 in the cardiology cohort (*p* = 0.002)). Similarly, high-intensity statins were significantly correlated with LDL-C control post-index (HR vs no statins: 4.23 in the primary care physician cohort (*p* < 0.0001), 3.07 in the diabetology cohort (*p* < 0.0001) and 3.45 (*p* < 0.0001) in the cardiology cohort). In addition to statins, treatment with PCSK9 inhibitors at index was also associated with a high probability of achieving LDL-C control (Supplemental Table 6). Among the CV risk factors or established CV conditions, diabetes mellitus was significantly associated with achieving LDL-C control in all three cohorts (HR: 1.45 in the primary care physician cohort (*p* < 0.0001), 1.77 in the diabetology cohort (*p* < 0.0001) and 1.59 in the cardiology cohort (*p* = 0.0094)). Similar results were observed for patients who had an LDL-C measurement at index that was at least 25% above their LDL-C goals (Supplemental Table 7).

## Discussion

The aim of this retrospective analysis was to assess the proportion of patients in Germany with uncontrolled hyperlipidaemia. For patients with uncontrolled LDL-C levels, the ‘distance to LDL-C goal’ (i.e. the degree to which LDL-C levels need to be reduced to achieve control) was classified. Results from this study demonstrate that LDL-C levels remain uncontrolled in a large proportion of patients. Most patients had uncontrolled LDL-C levels (⩾70 mg/dL; ⩾1.8 mmol/L) at index. In contrast to prior studies and registers, the ‘distance to LDL-C goal’ was assessed quantitatively. In each cohort, approximately one-third of patients required an LDL-C level reduction of 50%–100% relative to index to achieve their LDL-C goals. Notably, approximately one-tenth of patients required an LDL-C level reduction of approximately 150%–200% relative to index to reach their LDL-C goals. Similar results were observed in patients who were uncontrolled with an LDL-C measurement that was at least 25% above their LDL-C goals. This threshold was assigned as a measure of clinical relevance, based on the approximate 25% difference between the 2016 and 2019 ESC/EAS LDL-C targets (<70 and <55 mg/dL, respectively). Thus, most patients who had uncontrolled LDL-C at index required a substantial reduction in LDL-C levels to achieve control. Identifying the degree to which LDL-C levels need to be reduced to achieve control provides a clear picture of the high unmet medical need for patients with uncontrolled hyperlipidaemia who are at very high CV risk.

In this study, the proportions of patients not receiving any LLTs were high. In those receiving LLT, statin therapy was the most frequently administered, whereas the intensification of LLT (e.g. adding ezetimibe or PCSK9 inhibitors to statins) was low. Results from the regression analyses demonstrated that high-intensity treatments increased the probability of patients achieving LDL-C control. Patients treated with moderate-intensity or high-intensity statins were more likely to reach LDL-C control than those treated with low-dose statins, suggesting that the ability of statins to reduce LDL-C levels was dose-dependent among patients who were uncontrolled at index. These findings support the results of previous retrospective studies^21,22^ and suggest that statin intensification is associated with improved goal attainment, which could lead to improved CV outcomes. They also provide further evidence that optimization of statin regimens and the intensification of LLT may be required for patients to achieve their LDL-C goals. These results have important implications for clinical care and will drive the need for further treatment intensification in these patients.

Kaplan–Meier analyses were conducted to determine the time-to-LDL-C control among patients with uncontrolled LDL-C levels at the index date. Notably, LDL-C control was not achieved at 36 months post-index in most patients across all cohorts. These results are perhaps unsurprising, given that the proportions of patients not receiving any LLT in this study were high. However, these results suggest that LLTs are not being utilized to their full extent, or that patients may not have access to treatment options that lower LDL-C levels. Of note, there was a lower prevalence of LLT use at baseline in the diabetology and cardiology cohorts than in the primary care physician cohort. This finding is unexpected because LLTs must be initiated by a specialist or a doctor with specialist training. These data may suggest that diabetologists are the main specialists initiating treatment with LLTs and monitoring cholesterol levels, whereas cardiologists appear to primarily focus on heart-related interventions and surgeries and therefore are not the main prescribers. These data may also suggest gaps in the referral of patients to specialists, in particular, cardiologists. Of note, after initiation of LLTs by a specialist, patients are generally referred to their primary care physician for LDL-C monitoring and further prescriptions, which may also explain the lower prevalence of LLT use at baseline in the diabetology and cardiology cohorts than in the primary care physician cohort. Without the proper utilization of LLTs, many patients remain at an increased risk of experiencing severe CV events.^23^ Thus, there is an unmet need to optimize LLT in patients to lower LDL-C levels. It would be of interest in future studies to examine whether the patients with the greatest ‘distance to LDL-C goal’ initially had a later onset of LLT intensification than those who were close to achieving their LDL-C goal. In line with this, data from logistic regression analyses found a clear association between LLT intensification and LDL-C control. This association was greatest among patients in the primary care physician cohort, in whom statin therapy was significantly associated with achieving LDL-C control at index in a dose-dependent manner. Patients in the cardiology cohort who received PCSK9 inhibitors in addition to high-intensity statins were more likely to achieve LDL-C control than those only receiving high-intensity statins; however, the patient number in this cohort was small and this association was not statistically significant. Notably, a significant association between PCSK9 inhibitors and LDL-C control after index was observed; however, the small proportion of patients receiving PCSK9 inhibitors in each cohort make it difficult to draw definitive conclusions from these data. The efficacy of PCSK9 inhibitors, however, has been well established in previous clinical studies.^13,15^

The present study predominantly comprised older patients, with a mean age of approximately 70 years. Notably, at least 40% of these patients were receiving an LLT at index, despite their very high CV risk. In patients over 75 years old, prescribing LLTs must be carefully balanced against factors such as general physiological state, frailty, comorbidities, estimated CV risk, life expectancy and patient preferences. Additionally, it is important to consider that the relationship between hypercholesterolaemia, CV events and mortality tends to weaken in older populations. Therefore, a more individualized approach to lipid management may be required in elderly patients.

Our results are consistent with previously published studies in other European countries. A study in Italy spanning 2013–2018 reported that 88.2% of patients with very high CV risk had uncontrolled LDL-C levels at baseline, and the average reduction of LDL-C levels needed to meet the LDL-C goal was 44.0% for patients at very high CV risk.^24^ More recent baseline data from the SANTORINI study of patients at high or very high CV risk across 14 European countries in 2020–2021 found that LDL-C goal attainment was poor; 73.3% of patients had not achieved the 2019 ESC/EAS LDL-C goal.^25^ ‘Distance to LDL-C goal’ among these patients was not reported, but only 21.8% of patients had no documented LLT use, indicating higher LLT use than in the current study. This may account for the slightly higher proportion of patients who had achieved the 2019 ESC/EAS LDL-C goal.

Although PCSK9 inhibitor therapy is effective for reducing LDL-C levels,^13^ the use of PCSK9 inhibitors in patients with hyperlipidaemia is limited. Strict conditions limit the use of PCSK9 inhibitors across Europe; in Germany, the prescription of PCSK9 inhibitors is limited by a prescription restriction (‘Arzneimittel verordnungseinschränkung’) to specific high-risk populations. A prospective registry analysis showed that the levels of LDL-C at evolocumab initiation were nearly three times higher than the recommended European threshold for initiation of PCSK9 inhibitors, even though treatment was associated with improved outcomes.^13^ Increasing awareness of the efficacy of PCSK9 inhibitors and identifying the appropriate patients who can benefit from treatment may help to reduce the burden of hyperlipidaemia and associated CV risk factors or diseases.

The limitations of this study should be acknowledged. The potential for fragmentation of patient histories (due to the retrospective patient data used for analysis) and the unknown patient adherence to LLTs were the main limitations of this study. In the IQVIA Disease Analyzer database, it is not possible to track patients across physician specialities; therefore, it was not possible to capture previous LLT intensification that a patient may have received through a previous primary care physician or specialist. It was also not possible to capture when a patient may have switched from a primary care physician to a specialist. Consequently, patients who were treated by both a primary care physician and a specialist (i.e. a cardiologist or internal medicine specialist with an interest in diabetes or endocrinology) appear as two distinct patients, and this potential duplication of patients could skew the data. Incomplete patient histories could mean that CV risk factors or established CV conditions were not fully documented. Furthermore, incomplete patient histories could have contributed to the low LLT use recorded among patients in the cardiology cohort. Given that the study period covered January 2013 to September 2021, the data may not fully reflect the current situation due to the inclusion of data before the implementation of the 2019 ESC/EAS guidelines. However, the collection of data over such a long period is of value because these data strengthen the evidence that most patients require a substantial reduction of LDL-C levels to achieve their LDL-C goals, which remains a therapeutic challenge. Additionally, the study period covers a time period when some of the more recently approved LLTs (for example, bempedoic acid or inclisiran) were not yet available. Given that widespread adoption of newer compounds in clinical practice often takes some time, it was not possible to assess the ‘distance to LDL-C goal’ in patients receiving these LLTs. However, the data described here are still representative of current clinical practice as the therapies included in this study remain in use today with similar treatment algorithms. Newer LLTs may have a role to play in the future in achieving further reductions in LDL-C levels needed to reach LDL-C goals.^26–28^ Future analyses should examine the ‘distance to LDL-C goal’ with incorporation of data on the most recently approved LLTs. Another limitation is that, given the real-world observational nature of the study, the frequency of LDL-C measurements was not specified and probably varied. In clinical practice, LDL-C measurements are often only taken when clinicians are deciding on a treatment option or monitoring the effect of a new treatment, and this may have introduced some outcome ascertainment bias. For this study, the assessment of statin intolerance was based on the available data; patients who had received statins before index but no longer received them after index were considered statin intolerant. Therefore, future studies are warranted to assess the potential role of statin intolerance in LDL-C control.

## Conclusion

In conclusion, results from this study highlight that LDL-C levels remain uncontrolled in a large proportion of patients with hyperlipidaemia who have CV risk factors or established CV conditions. Analysis of the ‘distance to LDL-C goal’ among patients uncontrolled at index showed that most patients required a substantial reduction of LDL-C levels to achieve their LDL-C goals. Importantly, optimization of statin regimens and the intensification of LLT may be required for patients to achieve their LDL-C goals. Furthermore, increasing awareness of hyperlipidaemia and treatment options among primary care physicians and specialists may help to improve LDL-C control and reduce the ‘distance to LDL-C goal’.

## Supplemental Material

sj-docx-1-tak-10.1177_17539447241277402 – Supplemental material for Quantifying the ‘distance to LDC-goal’ in patients at very high cardiovascular risk with hyperlipidaemia in Germany: a retrospective claims database analysis Supplemental material, sj-docx-1-tak-10.1177_17539447241277402 for Quantifying the ‘distance to LDC-goal’ in patients at very high cardiovascular risk with hyperlipidaemia in Germany: a retrospective claims database analysis by Ksenija Stach, Hartmut Richter, Uwe Fraass and Alexandra Stein in Therapeutic Advances in Cardiovascular Disease

## Declarations

**Ethics approval and consent to participate:** Given that the study was based on retrospective de-identified data, ethical approval was not required.

**Consent for publication:** Not applicable.

**Availability of data and materials:** Data will be available on reasonable request from the corresponding author. Complete details are available at the following: https://wwwext.amgen.com/science/clinical-trials/clinical-data-transparency-practices/clinical-trial-data-sharing-request.
