# Corrigendum to “Quantifying the ‘distance to LDL-C goal’ in patients at very high cardiovascular risk with hyperlipidaemia in Germany: a retrospective claims database analysis”

**DOI:** 10.1177/17539447251333039

**Published:** 2025-04-02

Stach K, Richter H, Fraass U, Stein A. Quantifying the ‘distance to LDL-C goal’ in patients at very high cardiovascular risk with hyperlipidaemia in Germany: a retrospective claims database analysis. *Ther Adv Cardiovasc Dis*. 2024;18. doi:10.1177/17539447241277402

In the above-mentioned article, the title has been corrected from “Quantifying the ‘distance to LDC-goal’ in patients at very high cardiovascular risk with hyperlipidaemia in Germany: a retrospective claims database analysis” to “Quantifying the ‘distance to LDL-C goal’ in patients at very high cardiovascular risk with hyperlipidaemia in Germany: a retrospective claims database analysis”.

The article has been updated to reflect the correction.

