# Supplementary material for: Quantifying the ‘distance to LDC-goal’ in patients at very high cardiovascular risk with hyperlipidaemia in Germany: a retrospective claims database analysis
Source: Ther Adv Cardiovasc Dis. 2024 Sep 28;18:17539447241277402. doi: 10.1177/17539447241277402 (PMC11440620; doi:10.1177/17539447241277402)
Supplement: sj-docx-1-tak-10.1177_17539447241277402 – Supplemental material for Quantifying the ‘distance to LDC-goal’ in patients at very high cardiovascular risk with hyperlipidaemia in Germany: a retrospective claims database analysis [file sj-docx-1-tak-10.1177_17539447241277402.docx]

**Supplementary material**

**Supplementary Table 1.** Additional baseline patient characteristics by specialty cohort.

| **Parameter** | **Primary care physician cohort** | | | **Diabetology cohort** | | | **Cardiology cohort** | | |
| --- | --- | --- | --- | --- | --- | --- | --- | --- | --- |
|  | **Overall** | **Controlled** | **Uncontrolled** | **Overall** | **Controlled** | **Uncontrolled** | **Overall** | **Controlled** | **Uncontrolled** |
| **Total patients** | 27,159 (100.0) | 2449 (9.0) | 24,710 (91.0) | 3873 (100.0) | 525 (13.6) | 3348 (86.4) | 1931 (100.0) | 116 (6.0) | 1815 (94.0) |
| **Patient health insurance** | | | | | | | | | |
| Statutory | 24,948 (91.9) | 2212 (90.3) | 22,736 (92.0) | 3558 (91.9) | 485 (92.4) | 3073 (91.8) | 1731 (89.6) | 95 (81.9) | 1636 (90.1) |
| Private | 2211 (8.1) | 237 (9.7) | 1974 (8.0) | 315 (8.1) | 40 (7.6) | 275 (8.2) | 200 (10.4) | 21 (18.1) | 179 (9.9) |
| **Year of index date** | | | | | | | | | |
| 2014 | 7770 (28.6) | 623 (25.4) | 7147 (28.9) | 796 (20.6) | 85 (16.2) | 711 (21.2) | 837 (43.3) | 23 (19.8) | 814 (44.8) |
| 2015 | 6597 (24.3) | 619 (25.3) | 5978 (24.2) | 434 (11.2) | 43 (8.2) | 391 (11.7) | 329 (17.0) | 10 (8.6) | 319 (17.6) |
| 2016 | 6605 (24.3) | 579 (23.6) | 6026 (24.4) | 1523 (39.3) | 247 (47.0) | 1276 (38.1) | 340 (17.6) | 27 (23.3) | 313 (17.2) |
| 2017 | 3527 (13.0) | 337 (13.8) | 3190 (12.9) | 647 (16.7) | 95 (18.1) | 552 (16.5) | 226 (11.7) | 29 (25.0) | 197 (10.9) |
| 2018 | 2660 (9.8) | 291 (11.9) | 2369 (9.6) | 473 (12.2) | 55 (10.5) | 418 (12.5) | 199 (10.3) | 27 (23.3) | 172 (9.5) |
| **Cardiovascular diseases** | | | | | | | | | |
| Chronic ischaemic heart disease | 20,144 (74.2) | 1919 (78.4) | 18,225 (73.8) | 2658 (68.6) | 390 (74.3) | 2268 (67.7) | 1745 (90.4) | 105 (90.5) | 1640 (90.4) |
| Heart failure | 5940 (21.9) | 667 (27.2) | 5273 (21.3) | 806 (20.8) | 123 (23.4) | 683 (20.4) | 398 (20.6) | 36 (31.0) | 362 (19.9) |
| Atherosclerosis | 2879 (10.6) | 261 (10.7) | 2618 (10.6) | 300 (7.7) | 45 (8.6) | 255 (7.6) | 157 (8.1) | 16 (13.8) | 141 (7.8) |
| Aortic aneurysm | 844 (3.1) | 83 (3.4) | 761 (3.1) | 100 (2.6) | 15 (2.9) | 85 (2.5) | 67 (3.5) | 4 (3.4) | 63 (3.5) |
| Acute heart failure | 445 (1.6) | 56 (2.3) | 389 (1.6) | 63 (1.6) | 4 (0.8) | 59 (1.8) | 26 (1.3) | 2 (1.7) | 24 (1.3) |
| **Cardiovascular risk factors** | | | | | | | | | |
| Diabetes mellitus | 11,660 (42.9) | 1266 (51.7) | 10,394 (42.1) | 2620 (67.6) | 415 (79.0) | 2205 (65.9) | 458 (23.7) | 53 (45.7) | 405 (22.3) |
| Chronic kidney disease | 4074 (15.0) | 416 (17.0) | 3658 (14.8) | 1010 (26.1) | 166 (31.6) | 844 (25.2) | 143 (7.4) | 10 (8.6) | 133 (7.3) |
| **Statin tolerance (ref: statin tolerant)** | | | | | | | | | |
| Statin intolerant | 436 (1.6) | 7 (0.3) | 429 (1.7) | 52 (1.3) | 6 (1.1) | 46 (1.4) | 153 (7.9) | 15 (12.9) | 138 (7.6) |

Data presented as n (%) unless stated otherwise.

**Supplementary Table 2.** LLT use at index.

|  | **Primary care physician cohort** | | | **Diabetology cohort** | | | **Cardiology cohort** | | |
| --- | --- | --- | --- | --- | --- | --- | --- | --- | --- |
| **Index LLT** | **Overall** | **Controlled** | **Uncontrolled** | **Overall** | **Controlled** | **Uncontrolled** | **Overall** | **Controlled** | **Uncontrolled** |
| No LLT | 10,843 (39.9) | 400 (16.3) | 10,443 (42.3) | 2194 (56.6) | 256 (48.8) | 1938 (57.9) | 1457 (75.5) | 90 (77.6) | 1367 (75.3) |
| Other^a^ | 191 (1.3) | 10 (0.4) | 181 (0.7) | 20 (0.5) | 1 (0.2) | 19 (0.6) | 7 (0.4) | 0 (0.0) | 7 (0.4) |
| Ezetimibe | 146 (0.5) | 9 (0.4) | 137 (0.6) | 23 (0.6) | 1 (0.2) | 22 (0.7) | 18 (0.9) | 2 (1.7) | 16 (0.9) |
| Ezetimibe plus other^a^ | 6 (0.0) | 0 (0.0) | 6 (0.0) | 0 (0.0) | 0 (0.0) | 0 (0.0) | 0 (0.0) | 0 (0.0) | 0 (0.0) |
| PCSK9 inhibitor | 4 (0.0) | 0 (0.0) | 4 (0.0) | 0 (0) | 0 (0.0) | 0 (0.0) | 7 (0.4) | 2 (1.7) | 5 (0.3) |
| Low-dose statin monotherapy^b^ | 1018 (3.7) | 63 (2.6) | 955 (3.9) | 111 (2.9) | 7 (1.3) | 104 (3.1) | 41 (2.1) | 0 (0.0) | 41 (2.3) |
| Moderate-intensity statin monotherapy^b^ | 11,979 (44.1) | 1443 (58.9) | 10,538 (42.6) | 1167 (30.1) | 188 (35.8) | 979 (29.2) | 276 (14.3) | 16 (13.8) | 260 (14.3) |
| High-intensity statin monotherapy^b^ | 2020 (7.4) | 342 (14.0) | 1678 (6.8) | 238 (6.1) | 46 (8.8) | 192 (5.7) | 72 (3.7) | 2 (1.7) | 70 (3.9) |
| Any statin plus other^a^ | 44 (0.2) | 4 (0.2) | 40 (0.2) | 8 (0.2) | 1 (0.2) | 7 (0.2) | 3 (0.2) | 0 (0.0) | 3 (0.2) |
| Any statin plus ezetimibe | 904 (3.3) | 177 (7.2) | 727 (2.9) | 110 (2.8) | 25 (4.8) | 85 (2.5) | 48 (2.5) | 4 (3.4) | 44 (2.4) |
| Any statin plus ezetimibe plus other^a^ | 2 (0.0) | 1 (0.0) | 1 (0.0) | 2 (0.1) | 0 (0.0) | 2 (0.1) | 2 (0.1) | 0 (0.0) | 2 (0.1) |
| Any statin plus PCSK9 inhibitor | 2 (0.0) | 0 (0.0) | 2 (0.0) | 0 (0.0) | 0 (0.0) | 0 (0.0) | 0 (0.0) | 0 (0.0) | 0 (0.0) |
| **Statin treatment** | | | | | | | | | |
| No statins | 11,190 (41.2) | 419 (17.1) | 10771 (43.6) | 2237 (57.8) | 258 (49.1) | 1979 (59.1) | 1489 (77.1) | 94 (81.0) | 1395 (76.9) |
| Low-intensity statin^b^ | 1087 (4.0) | 73 (3.0) | 1014 (4.1) | 116 (3.0) | 8 (1.4) | 108 (3.2) | 47 (2.4) | 0 (0.0) | 47 (2.6) |
| Moderate-intensity statin^b^ | 12,621 (46.5) | 1557 (63.6) | 11,064 (44.8) | 1248 (32.2) | 203 (38.7) | 1045 (31.2) | 313 (16.2) | 18 (15.5) | 295 (16.3) |
| High-intensity statin^b^ | 2261 (8.3) | 400 (16.3) | 1861 (7.5) | 272 (7.0) | 56 (10.7) | 216 (6.5) | 82 (4.2) | 4 (3.4) | 78 (4.3) |
| **Ezetimibe treatment** | | | | | | | | | |
| No ezetimibe | 26,101 (96.1) | 2262 (92.4) | 23,839 (96.5) | 3738 (96.5) | 499 (95.0) | 3239 (96.7) | 1863 (96.5) | 110 (94.8) | 1753 (96.6) |
| With ezetimibe | 1058 (3.9) | 187 (7.6) | 871 (3.5) | 135 (3.5) | 26 (5.0) | 109 (3.3) | 68 (3.5) | 6 (5.2) | 62 (3.4) |
| **PCSK9 inhibitor treatment** | | | | | | | | | |
| No PCSK9 inhibitor | 27,153 (100.0) | 2449 (100.0) | 24,704  (100.0) | 3873 (100.0) | 525  (100.0) | 3348  (100.0) | 1924  (99.6) | 114  (98.3) | 1810  (99.7) |
| With PCSK9 inhibitor | 6 (0.0) | 0 (0.0) | 6 (0.0) | 0 (0.0) | 0 (0.0) | 0 (0.0) | 7 (0.4) | 2 (1.7) | 5 (0.3) |
| **Other LLT^a^** | | | | | | | | | |
| No other LLT | 26,916 (99.1) | 2434 (99.4) | 24,482 (99.1) | 3843 (99.2) | 523 (99.6) | 3320 (99.2) | 1919 (99.4) | 116 (100.0) | 1803 (99.3) |
| With other LLT | 243 (0.9) | 15 (0.6) | 228 (0.9) | 30 (0.8) | 2 (0.4) | 28 (0.8) | 12 (0.6) | 0 (0.0) | 12 (0.7) |

Data presented as n (%) unless stated otherwise.

^a^Other = fibrates or bile acid sequestrants.

^b^Statin dose by product form: low-dose statin, fluva ≤ 40 mg, lova ≤ 20 mg, prava ≤ 20 mg, simva ≤ 10 mg; medium-dose statin, atorva ≤ 20 mg, fluva > 40 mg – ≤ 80 mg, lova > 20 mg – ≤ 80 mg, pitava ≤ 4 mg, prava > 20 mg – ≤ 80 mg, rosuva ≤ 10 mg, simva > 10 mg – ≤ 40 mg; high-dose statin, atorva > 20 mg, rosuva > 10 mg, simva > 40 mg.

LLT, lipid-lowering therapy; PCSK9, proprotein convertase subtilisin/kexin type 9.

**Supplementary Table 3.** LLT use post-index and days to reach LDL-C control.

|  | **Primary care physician cohort** | | | **Diabetology cohort** | | | **Cardiology cohort** | | |
| --- | --- | --- | --- | --- | --- | --- | --- | --- | --- |
|  | **N patients (%)** | **Total**  **days**  **covered** | **Days/ patient** | **N patients (%)** | **Total**  **days covered** | **Days/ patient** | **N patients (%)** | **Total days covered** | **Days/ patient** |
| **Total patients** | 24,230 (100.0) | NA | NA | 3297 (100.0) | NA | NA | 1694 (100.0) | NA | NA |
| **Patients without LLT** | 4785 (19.7) | NA | NA | 878 (26.6) | NA | NA | 708 (41.8) | NA | NA |
| **Patients with at least intermittent LLT** | 19,445 (80.3) | NA | NA | 2419 (73.4) | NA | NA | 986 (58.2) | NA | NA |
| **Summary of distinct LLT classes** | | | | | | | | | |
| No LLT | 22,266 | 10,441,984 | 469.0 | 3097 | 1,581,479 | 510.6 | 1654 | 1,224,747 | 740.5 |
| Other LLT^a^ | 345 (1.8) | 134,999 | 391.3 | 53 (2.2) | 20,775 | 392.0 | 12 (1.2) | 1560 | 130.0 |
| Ezetimibe | 772 (4.0) | 169,226 | 219.2 | 125 (5.2) | 30,184 | 241.5 | 101 (10.2) | 16,748 | 165.8 |
| Ezetimibe plus other | 26 (0.1) | 7280 | 280.0 | 2 (0.1) | 79 | 39.5 | 2 (0.2) | 696 | 348.0 |
| PCSK9 inhibitor | 18 (0.1) | 8010 | 445.0 | 7 (0.3) | 1480 | 211.4 | 18 (1.8) | 7322 | 406.8 |
| PCSK9 plus other | 0 (0.0) | 0 | 0 | 1 (0.0) | 37 | 37.0 | 0 (0.0) | 0 | 0 |
| PCSK9 plus ezetimibe | 3 (0.0) | 154 | 51.3 | 1 (0.0) | 12 | 12.0 | 1 (0.1) | 291 | 291.0 |
| PCSK9 plus ezetimibe plus other | 0 (0.0) | 0 (0.0) | 0 (0.0) | 0 (0.0) | 0 (0.0) | 0 (0.0) | 0 (0.0) | 0 (0.0) | 0 (0.0) |
| Low-intensity statin | 1578 (8.1) | 82,1290 | 520.5 | 194 (8.0) | 93,736 | 483.2 | 98 (9.9) | 21,153 | 215.8 |
| Moderate-intensity statin | 15,008 (77.2) | 8,649,001 | 576.3 | 1742 (72.0) | 91,1874 | 523.5 | 542 (55.0) | 153,719 | 283.6 |
| High-intensity statin | 4180 (21.5) | 1,879,128 | 449.6 | 597 (24.7) | 248,064 | 415.5 | 335 (34.0) | 74,441 | 222.2 |
| Any statin plus other | 160 (0.8) | 40473 | 253.0 | 24 (1.0) | 6015 | 250.6 | 8 (0.8) | 1726 | 215.8 |
| Any statin plus ezetimibe | 2247 (11.6) | 941770 | 419.1 | 294 (12.2) | 113047 | 384.5 | 185 (18.8) | 47968 | 259.3 |
| Any statin plus ezetimibe plus other | 27 (0.1) | 5046 | 186.9 | 5 (0.2) | 3005 | 601.0 | 3 (0.3) | 1071 | 357.0 |
| Any statin plus PCSK9 inhibitor | 9 (0.0) | 1663 | 184.8 | 1 (0.0) | 7 | 7.0 | 2 (0.2) | 40 | 20.0 |
| Any statin plus PCSK9 inhibitor plus other | 0 (0.0) | 0 (0.0) | 0 (0.0) | 0 (0.0) | 0 (0.0) | 0 (0.0) | 0 (0.0) | 0 (0.0) | 0 (0.0) |
| Any statin plus PCSK9 inhibitor plus ezetimibe | 4 (0.0) | 402 | 100.5 | 1 (0.0) | 19 | 19.0 | 3 (0.3) | 285 | 95.0 |
| Any statin plus PCSK9 inhibitor plus ezetimibe plus other | 0 (0.0) | 0 (0.0) | 0 (0.0) | 0 (0.0) | 0 (0.0) | 0 (0.0) | 0 (0.0) | 0 (0.0) | 0 (0.0) |
| **Summary of statins (± PCSK9, other LLT^a^)** | | | | | | | | | |
| Low-intensity statin | 1681 (8.6) | 876,396 | 521.4 | 203 (8.4) | 102 343 | 504.2 | 105 (10.6) | 24 846 | 236.6 |
| Moderate-intensity statin | 15 741 (81.0) | 9,206,474 | 584.9 | 1847 (76.4) | 978 197 | 529.6 | 613 (62.2) | 182 279 | 297.4 |
| High-intensity statin | 4595 (23.6) | 2,255,903 | 490.9 | 669 (27.7) | 295 227 | 441.3 | 373 (37.8) | 93 278 | 250.1 |
| **Summary of ezetimibe (± statins, PCSK9 other LLT^a^)** | | | | | | | | | |
| Ezetimibe | 2511 (12.9) | 1 123 878 | 447.6 | 344 (14.2) | 146,346 | 425.4 | 241 (24.4) | 67 059 | 278.3 |
| **Summary of PCSK9 inhibitor (± statins, other LLT^a^)** | | | | | | | | | |
| PCSK9 inhibitor | 24 (0.1) | 10,229 | 426.2 | 8 (0.3) | 1555 | 194.4 | 21 (2.1) | 7938 | 378.0 |
| **Summary of other LLT^a^ (± statins, PCSK9 inhibitor)** | | | | | | | | | |
| Other LLT^a^ | 401 (2.1) | 187 798 | 468.3 | 59 (2.4) | 29,911 | 507.0 | 18 (1.8) | 5053 | 280.7 |

Data presented as n (%) unless stated otherwise.

Percentages are calculated as a proportion of those patients with at least intermittent LLT.

^a^Other LLT = fibrates or bile acid sequestrants.LDL-C, low-density lipoprotein-cholesterol; LLT, lipid-lowering therapy; PCSK9, proprotein convertase subtilisin/kexin type 9.

**Supplementary Table 4.** Results of the logistic regression analysis for the odds of being controlled (Y/N) for the various covariate parameters.

|  | **Primary care physician cohort** | | **Diabetology cohort** | | **Cardiology cohort** | |
| --- | --- | --- | --- | --- | --- | --- |
| **Parameter** | **Odds ratio (95% CI)** | ***p* Value** | **Odds ratio (95% CI)** | ***p* Value** | **Odds ratio (95% CI)** | ***p* Value** |
| **Health insurance (ref: statutory)** | | | | | | |
| Private | 1.215  (1.048–1.407) | 0.0097 | 1.000  (0.698–1.433) | 0.9989 | 1.669  (0.91–3.06) | 0.098 |
| **Additional cardiovascular risk factors** | | | | | | |
| Chronic ischaemic heart disease | 1.004  (0.889–1.134) | 0.9503 | 1.150  (0.887–1.492) | 0.2911 | 1.409  (0.641–3.098) | 0.3937 |
| Heart failure | 1.314  (1.187–1.456) | < 0.0001 | 1.112  (0.877–1.411) | 0.3813 | 1.295  (0.814–2.059) | 0.2748 |
| Atherosclerosis | 0.943 (0.818–1.086) | 0.4147 | 1.154  (0.815–1.635) | 0.4186 | 1.078  (0.548–2.121) | 0.8286 |
| Aortic aneurysm | 0.900  (0.709–1.141) | 0.3837 | 1.057  (0.594–1.88) | 0.8507 | 0.653  (0.217–1.966) | 0.449 |
| Other acute ischaemic heart disease | 1.173  (0.874–1.573) | 0.2884 | 0.389  (0.138–1.092) | 0.073 | 0.813  (0.169–3.906) | 0.7965 |

CI, confidence interval; Ref, reference.

**Supplementary Table 5.** Results of the logistic regression analysis for the odds of being controlled (Y/N) for the various covariate parameters in those with an LDL-C measurement 25% above their LDL-C goals (sensitivity analyses).

|  | **Primary care physician cohort** | | **Diabetology cohort** | | **Cardiology cohort** | |
| --- | --- | --- | --- | --- | --- | --- |
| **Parameter** | **Odds ratio (95% CI)** | ***p* Value** | **Odds ratio (95% CI)** | ***p* Value** | **Odds ratio  (95% CI)** | ***p* Value** |
| **Sex (ref: male)** | | | | | | |
| Female | 0.634  (0.594–0.677) | < 0.0001 | 0.578  (0.494–0.675) | < 0.0001 | 0.552  (0.408–0.747) | < 0.0001 |
| **Age class (ref: > 60**–**70 years)** | | | | | | |
| 18–30 years | 1.580  (0.388–6.436) | 0.5235 | NA | NA | NA | NA |
| > 30–40 years | 0.750  (0.443–1.27) | 0.2847 | 0.777  (0.254–2.378) | 0.6589 | 1.137  (0.22–5.872) | 0.8783 |
| > 40–50 years | 0.847  (0.707–1.015) | 0.0715 | 1.512  (0.974–2.347) | 0.0655 | 0.800  (0.373–1.715) | 0.5668 |
| > 50–60 years | 0.930  (0.839–1.031) | 0.1664 | 0.958  (0.747–1.23) | 0.7375 | 0.890  (0.586–1.35) | 0.5832 |
| > 70–80 years | 1.161  (1.074–1.255) | 0.0002 | 1.287  (1.071–1.548) | 0.0072 | 1.209  (0.885–1.652) | 0.2341 |
| > 80 years | 1.424  (1.3–1.561) | <.0001 | 1.280  (1.026–1.598) | 0.0287 | 1.892  (1.223–2.927) | 0.0042 |
| **Health insurance (ref: statutory)** | | | | | | |
| Private | 1.143  (1.027–1.272) | 0.0145 | 1.157  (0.891–1.502) | 0.2749 | 1.618  (1.06–2.47) | 0.0256 |
| **Cardiovascular risk factors** | | | | | | |
| Myocardial infarction | 1.283  (1.183–1.391) | < 0.0001 | 1.360  (1.07–1.728) | 0.0119 | 1.562  (1.1–2.219) | 0.0127 |
| Ischaemic stroke | 1.168  (1.082–1.261) | < 0.0001 | 0.961  (0.796–1.16) | 0.6771 | 0.986  (0.703–1.382) | 0.9328 |
| Chronic ischaemic heart disease | 1.189  (1.092–1.294) | < 0.0001 | 1.227  (1.01–1.49) | 0.039 | 2.024  (1.157–3.54) | 0.0135 |
| Transient ischaemic attack | 0.987  (0.883–1.103) | 0.8151 | 0.893  (0.664–1.2) | 0.4511 | 0.821  (0.292–2.313) | 0.7094 |
| Peripheral arterial disease | 0.963  (0.889–1.043) | 0.3526 | 1.050  (0.883–1.249) | 0.5819 | 1.292  (0.868–1.922) | 0.2063 |
| Cardiac bypass | 1.004  (0.891–1.131) | 0.9518 | 1.193  (0.916–1.555) | 0.1903 | 1.243  (0.885–1.746) | 0.2103 |
| PCI (angioplasty) with stenting | 1.240  (1.111–1.384) | 0.0001 | 1.490  (1.19–1.865) | 0.0005 | 1.825  (1.386–2.403) | < 0.0001 |
| Heart failure | 1.192  (1.108–1.282) | < 0.0001 | 1.075  (0.896–1.29) | 0.4342 | 1.351  (1.001–1.823) | 0.0491 |
| Carotid artery stenosis | 1.113  (0.997–1.244) | 0.0576 | 1.020  (0.773–1.346) | 0.8889 | 0.931  (0.601–1.443) | 0.7484 |
| Angina pectoris | 0.950  (0.871–1.036) | 0.243 | 1.163  (0.892–1.516) | 0.2642 | 0.461  (0.348–0.612) | < 0.0001 |
| Atherosclerosis | 1.002  (0.909–1.104) | 0.9737 | 0.985  (0.751–1.292) | 0.9146 | 1.330  (0.844–2.097) | 0.2188 |
| Aortic aneurysm | 1.041  (0.885–1.224) | 0.6305 | 0.948  (0.607–1.479) | 0.8131 | 1.140  (0.606–2.144) | 0.6851 |
| Other acute ischaemic heart disease | 1.105  (0.888–1.374) | 0.3715 | 0.501  (0.265–0.948) | 0.0336 | 0.994  (0.377–2.622) | 0.991 |
| Diabetes mellitus | 1.429  (1.344–1.519) | < 0.0001 | 2.126  (1.792–2.521) | < 0.0001 | 2.115  (1.602–2.793) | <0.0001 |
| Chronic kidney disease | 1.047  (0.962–1.14) | 0.2855 | 1.098  (0.928–1.299) | 0.2743 | 0.770  (0.479–1.239) | 0.2821 |
| Metabolic syndrome | 0.934  (0.812–1.073) | 0.333 | 0.814  (0.573–1.157) | 0.252 | 0.813  (0.231–2.863) | 0.7474 |
| **Statin tolerance (ref: statin tolerant)** | | | | | | |
| Statin intolerant | 0.391  (0.245–0.622) | < 0.0001 | 1.328  (0.708–2.492) | 0.3769 | 1.323  (0.835–2.097) | 0.2332 |
| **Statin dosage at index (ref: no statin therapy)** | | | | | | |
| Low-intensity statins | 1.774  (1.501–2.096) | < 0.0001 | 0.741  (0.459–1.196) | 0.2194 | 0.257  (0.075–0.879) | 0.0304 |
| Moderate-intensity statins | 3.766  (3.499–4.052) | < 0.0001 | 1.792  (1.531–2.096) | < 0.0001 | 1.394  (0.995–1.953) | 0.0533 |
| High-intensity statins | 5.223  (4.686–5.821) | < 0.0001 | 1.991  (1.513–2.619) | < 0.0001 | 0.831  (0.432–1.6) | 0.5799 |
| **Ezetimibe inhibitor at index (ref: no ezetimibe inhibitor)** | | | | | | |
| With ezetimibe inhibitor | 1.704 (1.491–1.946) | < 0.0001 | 1.281  (0.884–1.857) | 0.1909 | 1.486  (0.79–2.793) | 0.2193 |
| **PCSK9 inhibitor at index (ref: no PCSK9 inhibitor)** | | | | | | |
| With PCSK9 inhibitor | 14.455  (2.44–85.643) | 0.0033 | NA | NA | 1.786  (0.322–9.915) | 0.5073 |
| **Other LLT^a^ at index (ref: no other LLT)** | | | | | | |
| With other LLT^a^ | 0.998  (0.687–1.448) | 0.9897 | 0.767 (0.327–1.800) | 0.5427 | 1.472  (0.302–7.178) | 0.6323 |

^a^Other LTT = ezetimibe, fibrates, or bile acid sequestrants.

CI, confidence interval; LDL-C, low-density lipoprotein-cholesterol; LLT, lipid-lowering therapy; NA, not available; PCSK9, proprotein convertase subtilisin/kexin type 9; PCI, percutaneous coronary intervention; Ref, reference.

**Supplementary Table 6.** Cox regression analysis of the relationship between the time-to-LDL-C control and various covariates.

|  | **Primary care physician cohort** | | **Diabetology cohort** | | **Cardiology cohort** | |
| --- | --- | --- | --- | --- | --- | --- |
| **Parameter** | **Hazard ratio  (95% CI)** | ***p* Value** | **Hazard ratio  (95% CI)** | ***p* Value** | **Hazard ratio  (95% CI)** | ***p* Value** |
| **Sex (ref: male)** | | | | | | |
| Female | 0.639 (0.589–0.693) | < 0.0001 | 0.647 (0.538–0.779) | < 0.0001 | 0.453 (0.295–0.695) | 0.0003 |
| **Age class (ref: > 60–70 years)** | | | | | | |
| 18–30 years | 0.000 (0–>9999) | 0.9027 | NA (NA–NA) | NA | NA (NA–NA) | NA |
| > 30–40 years | 0.916 (0.504–1.663) | 0.7732 | 0.348 (0.048–2.501) | 0.2943 | 0 (0–>9999) | 0.9816 |
| > 40–50 years | 0.992 (0.812–1.213) | 0.9410 | 0.987 (0.568–1.718) | 0.9644 | 1.885 (0.915–3.883) | 0.0857 |
| > 50–60 years | 0.923 (0.82–1.039) | 0.1866 | 1.047 (0.794–1.382) | 0.7449 | 1.165 (0.69–1.966) | 0.5679 |
| > 70–80 years | 1.008 (0.919–1.106) | 0.8618 | 1.074 (0.872–1.322) | 0.5031 | 1.375 (0.896–2.111) | 0.1447 |
| > 80 years | 1.138 (1.017–1.274) | 0.0244 | 1.031 (0.793–1.34) | 0.8220 | 2.025 (1.126–3.644) | 0.0185 |
| **Health insurance (ref: statutory)** | | | | | | |
| Private | 1.017 (0.892–1.159) | 0.7981 | 1.16 (0.86–1.564) | 0.3316 | 0.963 (0.563–1.648) | 0.8909 |
| **Risk factors indicating clinical manifestation of atherosclerosis (in each case, ref: no)** | | | | | | |
| Myocardial infarction | 1.100 (1.003–1.206) | 0.042 | 0.893 (0.674–1.183) | 0.4301 | 0.978 (0.626–1.527) | 0.9217 |
| Ischaemic stroke | 1.162 (1.065–1.268) | 0.0007 | 1.098 (0.896–1.346) | 0.3686 | 0.840 (0.554–1.274) | 0.4109 |
| Chronic ischaemic heart disease | 1.346 (1.208–1.5) | < 0.0001 | 1.238 (0.989–1.55) | 0.0622 | 1.098 (0.552–2.183) | 0.7897 |
| Transient ischaemic attack | 1.081 (0.951–1.23) | 0.2334 | 1.061 (0.779–1.446) | 0.7054 | 1.431 (0.591–3.466) | 0.4271 |
| Peripheral arterial disease | 1.102 (1.009–1.204) | 0.031 | 1.262 (1.045–1.525) | 0.0156 | 1.654 (1.062–2.576) | 0.0262 |
| Cardiac bypass | 1.077 (0.95–1.221) | 0.2455 | 1.017 (0.768–1.347) | 0.9058 | 1.129 (0.741–1.722) | 0.5716 |
| PCI (angioplasty) with stenting | 1.256 (1.122–1.406) | < 0.0001 | 1.087 (0.845–1.397) | 0.5174 | 1.411 (0.992–2.007) | 0.0558 |
| Heart failure | 1.176 (1.083–1.278) | < 0.0001 | 1.220 (1.002–1.485) | 0.0477 | 1.478 (1.029–2.123) | 0.0345 |
| Carotid artery stenosis | 1.062 (0.941–1.199) | 0.3324 | 1.157 (0.876–1.528) | 0.3041 | 1.508 (0.968–2.35) | 0.0695 |
| Angina pectoris | 0.940 (0.852–1.037) | 0.2181 | 1.088 (0.821–1.442) | 0.5579 | 0.524 (0.363–0.758) | 0.0006 |
| Atherosclerosis | 1.058 (0.951–1.177) | 0.3021 | 0.977 (0.734–1.301) | 0.8751 | 1.002 (0.574–1.75) | 0.9931 |
| Aortic aneurysm | 0.956 (0.8–1.142) | 0.6175 | 0.713 (0.417–1.22) | 0.2174 | 0.937 (0.399–2.201) | 0.8806 |
| Other acute ischaemic heart diseases | 1.059 (0.836–1.341) | 0.6343 | 1.215 (0.707–2.087) | 0.4816 | 1.904 (0.756–4.794) | 0.1715 |
| Diabetes mellitus | 1.448 (1.345–1.559) | < 0.0001 | 1.768 (1.438–2.174) | < 0.0001 | 1.586 (1.12–2.245) | 0.0094 |
| Chronic kidney disease | 1.185 (1.082–1.298) | 0.0003 | 1.192 (0.989–1.437) | 0.0648 | 1.006 (0.611–1.655) | 0.9818 |
| Metabolic syndrome | 0.951 (0.817–1.108) | 0.5208 | 0.987 (0.682–1.43) | 0.9463 | 0.456 (0.059–3.5) | 0.4501 |
| **Statin tolerance (ref: statin tolerant)** | | | | | | |
| Statin intolerant | 0.429 (0.138–1.334) | 0.1436 | 0 (0–>9999) | 0.957 | 2.056 (0.736–5.742) | 0.1692 |
| **Statin dosage at index (ref: no statin therapy)** | | | | | | |
| Low-intensity statins | 1.089 (0.843–1.406) | 0.5146 | 0.672 (0.344–1.313) | 0.2449 | 0.813 (0.197–3.35) | 0.7741 |
| Medium-intensity statins | 2.626 (2.393–2.881) | < 0.0001 | 2.021 (1.672–2.442) | < 0.0001 | 1.981 (1.278–3.071) | 0.0022 |
| High-intensity statins | 4.229 (3.774–4.739) | < 0.0001 | 3.073 (2.413–3.915) | < 0.0001 | 3.446 (2.175–5.461) | < 0.0001 |
| **PCSK9 inhibitor at index (ref: no PCSK9 inhibitor)** | | | | | | |
| With PCSK9 inhibitor | 5.577 (2.312–13.45) | < 0.0001 | 26.128 (8.056–84.742) | < 0.0001 | 6.142 (2.103–17.938) | 0.0009 |
| **Ezetimibe at index (ref: no ezetimibe)** | | | | | | |
| With ezetimibe | 2.850 (2.576–3.153) | < 0.0001 | 1.859 (1.405–2.46) | < 0.0001 | 2.692 (1.659–4.368) | < 0.0001 |
| **Other LLT^a^ at index (ref: no other LLT^a^)** | | | | | | |
| With other LLT^a^ | 1.114 (0.731–1.697) | 0.6154 | 0.646 (0.266–1.57) | 0.3344 | 0.000 (0–>9999) | 0.9912 |
| **Current year (ref: pre-2019, 70 mg/dL limit^b^)** | | | | | | |
| 2019 onwards (55 mg/dL LDL-C limit)^c^ | 0.306 (0.262–0.357) | < 0.0001 | 0.288 (0.203–0.41) | < 0.0001 | 0.447 (0.228–0.874) | 0.0187 |

^a^Other = fibrates or bile acid sequestrants.

^b^A LDL-C level of 70 mg/dL according to the 2016 ESC/EAS LDL-C guidelines.

^c^A LDL-C level of 55 mg/dL according to the 2019 ESC/EAS LDL-C guidelines.

CI, confidence interval; LDL-C, low-density lipoprotein-cholesterol; LLT, lipid-lowering therapy; PCI, percutaneous coronary intervention; PCSK9, proprotein convertase subtilisin/kexin type 9; Ref, reference.

**Supplementary Table 7.** Cox regression analysis of the relationship between the time-to-LDL-C control and various covariates (sensitivity analysis).

|  | **Primary care physician cohort** | | **Diabetology cohort** | | **Cardiology cohort** | |
| --- | --- | --- | --- | --- | --- | --- |
| **Parameter** | **Hazard ratio  (95% CI)** | ***p* Value** | **Hazard ratio  (95% CI)** | ***p* Value** | **Hazard ratio  (95% CI)** | ***p* Value** |
| **Sex (ref: male)** | | | | | | |
| Female | 0.721 (0.679–0.765) | < 0.0001 | 0.705 (0.609–0.817) | < 0.0001 | 0.622 (0.478–0.809) | 0.0004 |
| **Age class (ref: > 60–70 years)** | | | | | | |
| 18–30 years | 0.000 (0–>9999) | 0.8908 | NA (NA–NA) | NA | NA (NA–NA) | NA |
| > 30–40 years | 0.878 (0.558–1.382) | 0.7573 | 0.652 (0.241–1.763) | 0.3991 | 0.424 (0.058–3.119) | 0.3993 |
| > 40–50 years | 0.978 (0.84–1.139) | 0.7752 | 0.854 (0.531–1.372) | 0.5142 | 0.789 (0.427–1.459) | 0.4498 |
| > 50–60 years | 0.929 (0.85–1.016) | 0.1079 | 1.028 (0.824–1.281) | 0.8094 | 0.766 (0.531–1.105) | 0.1545 |
| > 70–80 years | 1.015 (0.947–1.088) | 0.6709 | 0.986 (0.829–1.172) | 0.8729 | 1.141 (0.865–1.504) | 0.3516 |
| > 80 years | 1.057 (0.968–1.153) | 0.2176 | 1.134 (0.92–1.397) | 0.2392 | 1.297 (0.855–1.968) | 0.2213 |
| **Health insurance (ref: statutory)** | | | | | | |
| Private | 1.022 (0.924–1.131) | 0.6699 | 1.166 (0.908–1.497) | 0.2278 | 1.220 (0.834–1.784) | 0.3065 |
| **Risk factors indicating clinical manifestation of atherosclerosis (in each case, ref: no)** | | | | | | |
| Myocardial infarction | 1.103 (1.026–1.186) | 0.0079 | 1.001 (0.793–1.264) | 0.9907 | 1.320 (0.976–1.786) | 0.0711 |
| Ischaemic stroke | 1.166 (1.091–1.247) | < 0.0001 | 0.959 (0.809–1.137) | 0.6283 | 1.143 (0.862–1.516) | 0.3522 |
| Chronic ischaemic heart disease | 1.286 (1.188–1.393) | < 0.0001 | 0.964 (0.806–1.153) | 0.6886 | 1.551 (0.951–2.531) | 0.0788 |
| Transient ischaemic attack | 1.036 (0.938–1.145) | 0.4837 | 1.222 (0.965–1.548) | 0.0959 | 1.504 (0.811–2.789) | 0.1952 |
| Peripheral arterial disease | 1.073 (1.002–1.149) | 0.0435 | 1.226 (1.046–1.438) | 0.012 | 1.083 (0.762–1.538) | 0.6582 |
| Cardiac bypass | 1.045 (0.944–1.155) | 0.3968 | 0.980 (0.764–1.256) | 0.8704 | 1.534 (1.147–2.05) | 0.0039 |
| PCI (angioplasty) with stenting | 1.233 (1.124–1.353) | < 0.0001 | 1.266 (1.023–1.568) | 0.0304 | 1.199 (0.932–1.543) | 0.1574 |
| Heart failure | 1.073 (1.005–1.146) | 0.0354 | 1.268 (1.076–1.494) | 0.0046 | 1.295 (0.997–1.683) | 0.0528 |
| Carotid artery stenosis | 1.051 (0.955–1.156) | 0.3095 | 0.980 (0.767–1.251) | 0.8705 | 1.822 (1.368–2.428) | < .0001 |
| Angina pectoris | 1.005 (0.932–1.083) | 0.8983 | 0.938 (0.732–1.201) | 0.6107 | 0.732 (0.57–0.942) | 0.0151 |
| Atherosclerosis | 1.069 (0.984–1.162) | 0.1138 | 0.961 (0.762–1.212) | 0.7359 | 1.290 (0.889–1.871) | 0.1801 |
| Aortic aneurysm | 0.954 (0.828–1.1) | 0.5201 | 1.322 (0.932–1.876) | 0.1180 | 1.121 (0.65–1.936) | 0.6811 |
| Other acute ischaemic heart diseases | 1.043 (0.863–1.261) | 0.6643 | 0.948 (0.589–1.526) | 0.8258 | 0.948 (0.385–2.333) | 0.9070 |
| Diabetes mellitus | 1.328 (1.255–1.404) | < 0.0001 | 1.500 (1.28–1.757) | < 0.0001 | 1.310 (1.019–1.683) | 0.0351 |
| Chronic kidney disease | 1.095 (1.018–1.177) | 0.0152 | 1.148 (0.983–1.342) | 0.0818 | 1.110 (0.79–1.56) | 0.547 |
| Metabolic syndrome | 1.062 (0.946–1.193) | 0.3079 | 0.859 (0.626–1.179) | 0.3477 | 0.769 (0.234–2.535) | 0.6666 |
| **Statin dosage at index (ref: no statin therapy)** | | | | | | |
| Low–intensity statins | 1.608 (1.364–1.896) | < 0.0001 | 1.236 (0.821–1.861) | 0.311 | 1.044 (0.46–2.373) | 0.9172 |
| Moderate–intensity statins | 3.119 (2.912–3.34) | < 0.0001 | 2.278 (1.95–2.662) | < 0.0001 | 1.524 (1.099–2.115) | 0.0116 |
| High–intensity statins | 4.698 (4.304–5.128) | < 0.0001 | 4.023 (3.302–4.902) | < 0.0001 | 3.799 (2.789–5.174) | < 0.0001 |
| **Ezetimibe at index (ref: no ezetimibe)** | | | | | | |
| With ezetimibe | 2.646 (2.434–2.877) | < 0.0001 | 1.603 (1.245–2.063) | 0.0002 | 1.672 (1.119–2.498) | 0.012 |
| **PCSK9 inhibitor at index (ref: no PCSK9)** | | | | | | |
| With PCSK9 inhibitor | 18.281 (9.452–35.355) | < 0.0001 | 19.107 (4.609–79.215) | < .0001 | 7.680 (3.684–16.012) | < .0001 |
| **Other LLT^a^ at index (ref: no other LLT^a^)** | | | | | | |
| With other LLT^a^ | 1.230 (0.919–1.646) | 0.1646 | 0.577 (0.272–1.224) | 0.1517 | 0.000 (0–>9999) | 0.9663 |
| **Current year (ref: pre–2019 – 70 mg/dL limit)** | | | | | | |
| 2019 onwards (55 mg/dL LDL-C limit)^b^ | 0.343 (0.305–0.385) | < 0.0001 | 0.324 (0.245–0.427) | < .0001 | 0.321 (0.181–0.57) | 0.0001 |
| **Statin tolerance (ref: statin tolerant)** | | | | | | |
| Statin intolerant | 0.259 (0.097–0.691) | 0.007 | 0.000 (0–>9999) | 0.9404 | 1.132 (0.491–2.608) | 0.7708 |

CI, confidence interval; LLT, lipid lowering therapy; PCI, percutaneous coronary intervention; PCSK9, proprotein convertase subtilisin/kexin type 9; Ref, reference.

**Supplementary Figure 1.** Kaplan–Meier curve for time-to-LLT escalation among patients who had not achieved LDL-C control at index, overall, and by number of CV risk factors or CV diseases in the (a) primary care physician cohort, (b) diabetology cohort, and (c) cardiology cohort.


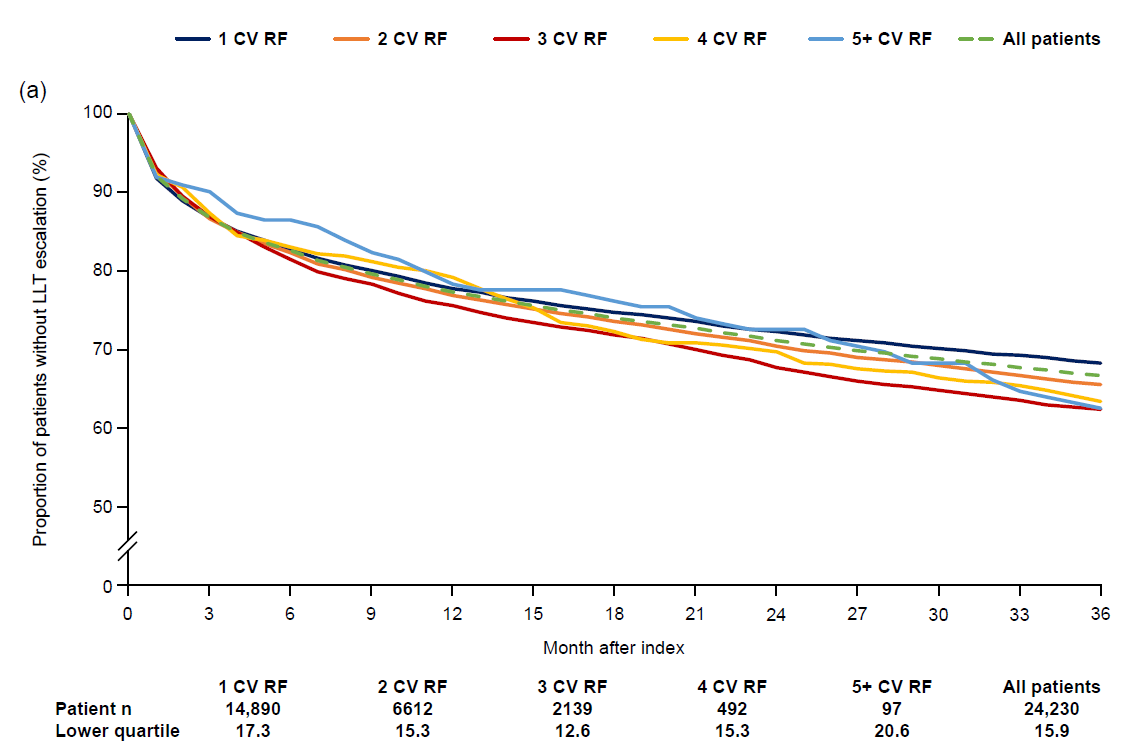


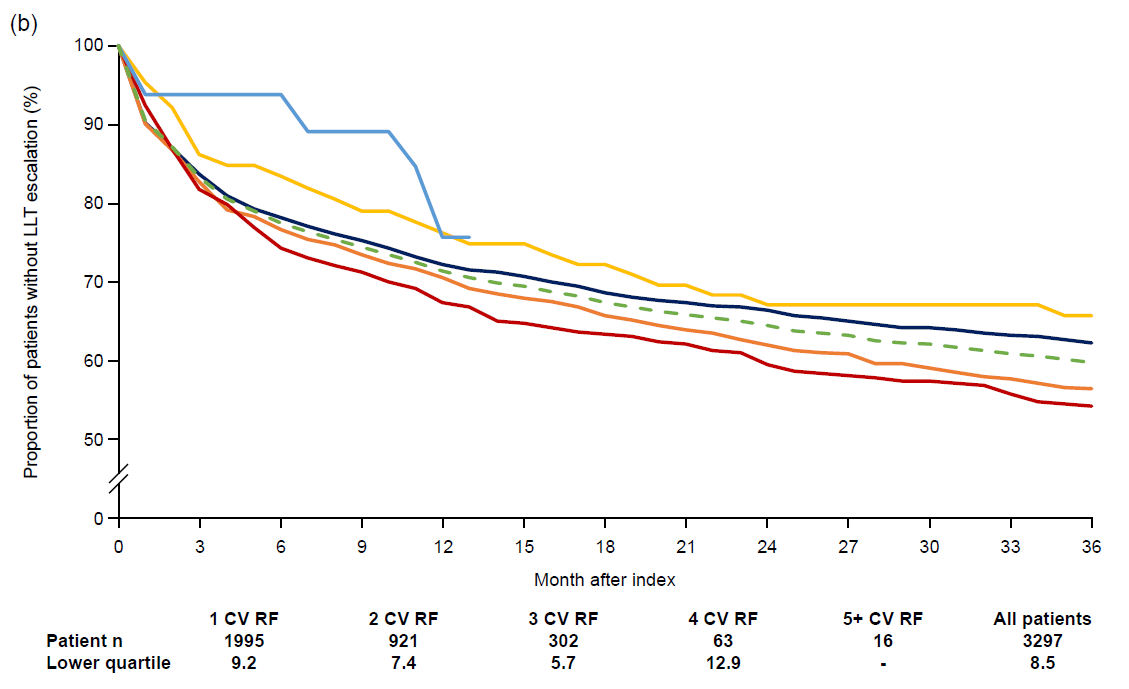


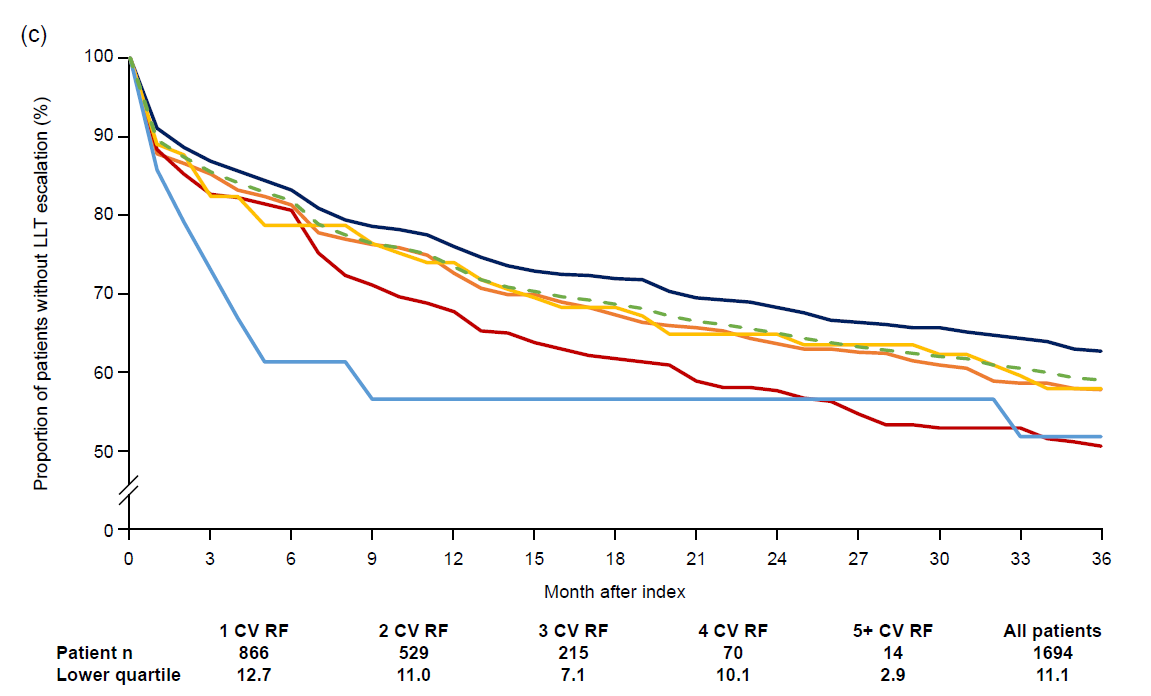


CV, cardiovascular; LDL-C, low-density lipoprotein-cholesterol; LLT, lipid-lowering therapy; RF risk factor.
